# Potential Mechanisms of Yiqi Jiedu Huayu Decoction in the Treatment of Diabetic Microvascular Complications Based on Network Analysis, Molecular Docking, and Experimental Validation

**DOI:** 10.1155/2023/5034687

**Published:** 2023-02-10

**Authors:** Chen Xuan, Weisen Ding, Ling Zhan, Yanying Xiong, Xiao Yu, Wenfu Cao, Yan Luo

**Affiliations:** ^1^Department of Fundamental Medicine, Chengdu University of Traditional Chinese Medicine, Chengdu 611137, China; ^2^College of Traditional Chinese Medicine, Chongqing Medical University, Chongqing 400016, China; ^3^Chongqing Traditional Chinese Medicine Hospital, Chongqing 400021, China

## Abstract

**Background:**

Diabetic microvascular complications are the main causes of organ dysfunction and even death in diabetic patients. Our previous studies confirmed the beneficial effects of Yiqi Jiedu Huayu Decoction (YJHD) on diabetic cardiomyopathy and diabetic nephropathy. It is not clear whether YJHD can treat multiple diabetic microvascular complications including diabetic retinopathy, diabetic cardiomyopathy, and diabetic nephropathy through some common mechanisms.

**Methods:**

TCMSP, SymMap, STITCH, Swiss Target Prediction, and SEA databases were used to collect and analyze the components and targets of YJHD. GeneCards, DrugBank, DisGeNET, OMIM, and GEO databases were used to obtain target genes for diabetic retinopathy, diabetic cardiomyopathy, and diabetic nephropathy. The GO and KEGG enrichment analyses were performed on the DAVID and STRING platforms. Molecular docking was used to evaluate the binding sites and affinities of compounds and target proteins. Animal experiments were designed to validate the network pharmacology results.

**Results:**

Through network pharmacological analysis, oxidative stress, inflammatory response, and apoptosis were identified as key pathological phenotypes for the treatment of diabetic microvascular complications with YJHD. In addition, JNK, p38, and ERK1/2 were predicted as key targets of YJHD in regulating the abovementioned pathological phenotypes. The results of animal experiments showed that YJHD could ameliorate retinal pathological changes of diabetes rats. YJHD can inhibit oxidative stress and inflammation in heart and kidney of diabetic rats. Molecular docking showed strong binding between compounds and JNK, p38, and ERK1/2. Berlambine may play a key role in the treatment process and is considered as a promising regulator of MAPK protein family. The regulatory effects of YJHD on JNK, p38, and ERK1/2 were demonstrated in animal experiments.

**Conclusions:**

YJHD may play a therapeutic role in diabetic microvascular complications by regulating oxidative stress, inflammatory response, and apoptosis. The regulation of JNK, p38, and ERK1/2 phosphorylation may be the key to its therapeutic effect.

## 1. Introduction

As a disease characterized by hyperglycemia, diabetes is a great threat to human health. According to the International Diabetes Federation, approximately 463 million adults suffered from diabetes in 2019. Without effective control, this data will grow by 51% in 2045 to about 700 million people [1]. Diabetic microvascular complications are one of the main causes of organ dysfunction and even death in diabetic patients [2]. Long-term hyperglycemia can damage the structure and function of the eyes, heart, and kidneys, leading to diabetic microvascular complications [3, 4]. Diabetic retinopathy (DR), diabetic cardiomyopathy (DCM), and diabetic nephropathy (DN) are three major diabetic microvascular complications.

DR is a common microvascular complication of diabetes. Approximately 80% of all patients with diabetes will eventually develop to DR. Severe DR is often associated with irreversible retinal damage and even blindness [5, 6]. Approximately 4.2 million patients died of diabetes in 2019 alone. DCM and DN are the main causes of death in diabetic patients [7]. DCM affects approximately 70% of patients with diabetes. Pathological changes in DCM include myocardial fibrosis, left ventricular hypertrophy, and impaired left ventricular systolic and diastolic function. Without effective treatment, these pathological changes will eventually lead to cardiac dysfunction, heart failure, and even death [8, 9]. At least about 30% of diabetic patients suffer from DN [10]. DN is the leading cause of end-stage renal disease (ESRD). When renal disease progresses to the ESRD stage, it tends to mean irreversible damage to the kidney [11]. Patients with diabetic microvascular complications often have to bear serious consequences if they are not treated promptly and effectively. Unfortunately, the microvascular complications of diabetes involve multiple organs and tissues, the pathogenesis of which remains unclear, and lack of effective and specific therapeutic drugs. Therefore, it is particularly important to develop an early and effective treatment for diabetic complications. In recent years, more and more studies have shown that Chinese herbs and their compounds may be the potential candidates for the treatment of diabetic microvascular complications.

Over the years, Yiqi Jiedu Huayu Decoction (YJHD) has been used to treat diabetic microvascular complications with reliable results. As a Chinese herbal formula, YJHD consists of seven different herbs, including HuangQi (*Astragalus mongholicus* Bunge), GeGen (*Pueraria montana var*. lobata (Willd.) Maesen and S. M. Almeida ex Sanjappa and Predeep), HuangQin (*Scutellaria baicalensis* Georgi), HuangLian (*Coptis chinensis* Franch), SanLeng (*Sparganium stoloniferum* (Buch.-Ham. ex Graebn.) Buch.-Ham. ex Juz), JiangHuang (*Curcuma longa* L), and SangYe (*Morus alba* L). Our previous results showed that YJHD was effective in improving cardiac and renal damage in diabetic rats [12, 13]. By regulating the balance of the PI3K/Akt and AMPK signaling pathways and promoting autophagy, YJHD can ameliorate renal injury and protect renal function [12]. In the heart tissue of diabetic rats, YJHD can suppress the high expression of TNF-*α*, NF-*κ*B, IL-1*β,* and IL-6 and ameliorate the heart injury by alleviating inflammatory response [13]. However, it remains unclear whether YJHD has the same therapeutic effect on DR. In this study, we focused on the common mechanisms involved in multiple diabetic microvascular complications, and the role of YJHD in these common mechanisms.

**Figure 1:**
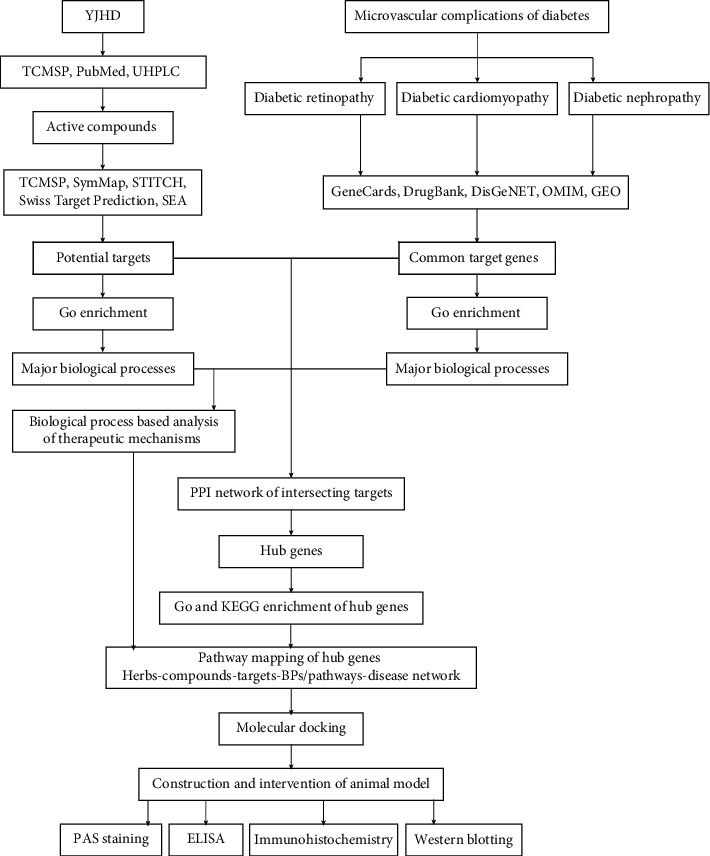
Overall protocol for this study.

The aim of this study is to elucidate the potential mechanisms of YJHD against multiple diabetic microvascular complications. For the purpose of this study, network pharmacology analysis, molecular docking, and animal experiments were designed. The specific experimental process is shown in Figure 1.

## 2. Methods

### 2.1. Identification of Active Compounds in YJHD

As a systems pharmacology platform of Chinese herbal medicines, TCMSP provides ADME information of compounds, which facilitates researchers to screen compounds [14]. Among these information, Oral Bioavailability (OB) and Drug-Like (DL) are parameters of interest in Chinese herbal medicine studies. Diabetes microangiopathy is a chronic complication of diabetes. Half Life (HL) is an important parameter of drug for chronic diseases. In this study, OB ≥ 30%, DL ≥ 0.18, and HL > 4 were used as criteria for screening active compounds from TCMSP [15, 16]. In addition, some compounds, such as astragaloside IV, although not meeting the screening parameters in this study, have been confirmed to be associated with the pharmacological effects of herbs. To avoid the deviation from missing these compounds, the PubMed platform was used to collect these compounds [17]. In our previous study, 15 key compounds of YJHD were identified by ultrahigh performance liquid chromatography (UHPLC), and these compounds were also included in this study (Supplementary 1) [12].

### 2.2. Targets Prediction and Analysis of YJHD

TCMSP, SymMap, STITCH, Swiss Target Prediction, and SEA are five different comprehensive databases that predict, screen, and collect targets based on different underlying logic [14, 18–21]. To avoid bias introduced by using a single database, these five databases were simultaneously adopted in this study.

All targets of YJHD were input into STRING platform for their protein-protein interaction (PPI) information [22]. The network connectivity degree (Degree) of these targets was scored in Cytoscape software [23]. The part of targets with degree ≥2 times of the median of all targets was considered to play a core connecting role in the PPI network. These targets were extracted and analyzed by gene ontology (GO) enrichment based on DAVID platform [24].

### 2.3. Target Genes Collection and Analysis of Diabetic Microvascular Complications

Target genes of diabetic microvascular complications were also derived from five commonly used databases, including GeneCards, DrugBank, DisGeNET, OMIM, and GEO [25–29]. Each of these databases has its own characteristics and limitations. To avoid the limitations introduced by different databases, this study adopted a strategy of integrating the information of these databases. Different from the other four databases, GEO provides high-throughput sequencing results of retinal tissues of DR, heart tissues of DCM, and kidney tissues of DN. These results demonstrate the difference of gene expression between the tissues with diabetic microvascular complications and normal tissues. Venn diagram was used to obtain the intersection genes of DR, DCM, and DN. These genes were then imported into DAVID platform for GO enrichment.

### 2.4. Analysis of Therapeutic Mechanisms Based on BP

Biological process (BP) items of YJHD targets and diabetic microvascular complications target genes were ranked by -Log10 (p.adjust), respectively. The top 20 BP were extracted as the main biological processes. Next, the common BP of YJHD and diabetic microvascular complications were obtained by intersection. Based on these BP and targets, a Targets-BP network was constructed.

### 2.5. Construction and Analysis of Protein-Protein Interaction Networks

The targets of YJHD and the common target genes of DR, DCM, and DN were intersected to obtain potential therapeutic targets. These targets were extracted and input into STRING platform for PPI information. Based on the PPI information of targets, Cytoscape software was used to construct the PPI network and perform topological analysis. Similarly, targets with Degree ≥2 times the median of all targets were extracted as the core targets to construct the Hub gene database.

### 2.6. Enrichment Analysis Based on Hub Genes

Hub genes are the focus of this research on YJHD in the treatment of diabetic microvascular complications. In this part, the GO and Kyoto Encyclopedia of Genes and Genomes (KEGG) pathway enrichment were used to analyze Hub genes. DAVID is a comprehensive set of functional annotation tools, facilitating researchers' understanding of the biological significance behind a large number of genes [24]. In this study, Hub genes were input into DAVID platform for GO enrichment analysis. Similarly, STRING platform was used for KEGG enrichment analysis of hub genes. The number of genes enriched in signaling pathways was used to rank the KEGG pathways. To further analyze the role of genes in pathways, Hub genes were mapped in the KEGG pathways.

### 2.7. Molecular Docking

The affinity between compounds and target proteins is very important information for their pharmacological effects. Autodock 4 and Autodock Vina are two commonly used software for predicting binding site and binding energy of compounds to target proteins. Compared with Autodock 4, Autodock Vina has better docking accuracy and shorter docking time [30]. Therefore, Autodock Vina was used for molecular docking in this study. In the Herbs-Compounds-Hub genes-Pathways/BP-Disease network, the corresponding relationships between JNK1, p38, ERK1, ERK2, and compounds were extracted. Then, MOL2 format files of compounds and PDB format files of proteins were obtained from ZINC and RCSB PDB databases, respectively [31, 32]. Based on these files, Autodock Vina was used for molecular docking to acquire binding site and binding energy. Finally, the binding patterns of the most affinity compounds to proteins were visualized using Discovery Studio software.

### 2.8. Animal Experiments

Ten-week-old male Sprague–Dawley rats (250 ± 30 g) used in this study were obtained from the animal experimental center of Chongqing Medical University. These rats were kept in a specific-pathogen-free level animal room at animal experimental center of Chongqing Medical University (SYXK 2018–0003). The animal center provides an environment of constant temperature (21 ± 1°C), constant humidity (55 ± 5%), and 12 hour alternating light and dark. All rats were free to get enough food and water. This study was approved by the ethics committee of the First Affiliated Hospital of Chongqing Medical University (permit no. 2020–454). All experimental operations in this study were carried out in accordance with the guidelines for the care and use of laboratory animals of the National Institutes of health.

A total of 28 rats were randomly divided into three groups: normal control group (8), diabetic group (10), and YJHD group (10). To construct the diabetic rat models, streptozotocin dissolved in citric acid solution was injected into the peritoneal cavity of the rats in the diabetic and YJHD groups at a dose of 60 mg/kg [12]. In order to avoid the influence of citric acid solution on the experimental results, the rats in the normal control group received intraperitoneal injection with a corresponding volume of citric acid solution. One week after streptozotocin injection, the animal model was evaluated by detecting fasting blood glucose twice per rat. When both fasting blood glucose tests were greater than 16.7 mmol/l, the diabetic model was considered to be established [12]. The drug intervention was administered by gavage once a day for 12 weeks after the establishment of the diabetic rat model. In the clinical use of YJHD granules, the daily dose for adults is 11.5 g. According to the surface area conversion ratio between rats and humans (6.3), the daily dose of rats is calculated as 1.2 g/kg. The YJHD group rats were gavaged with YJHD granule aqueous solution, while the normal control group and diabetic group rats were gavaged with corresponding doses of pure water [12]. Two rats in each of the diabetic and YJHD groups died during the 12 weeks drug intervention.

At the end of drug intervention, all rats were sacrificed under anesthesia. The eyeballs, heart, kidneys, and blood of rats were extracted and stored under suitable conditions. The eyeballs were fixed in 4% paraformaldehyde solution. The retina was then stripped for PAS staining. Part of the heart and kidney tissues was fixed in 4% paraformaldehyde solution for subsequent immunohistochemical staining. Another part of the heart and kidney tissues was snap frozen by liquid nitrogen and transferred to a −80°C refrigerator for storage. The serum was extracted from blood samples and stored in a −80°C refrigerator.

ELISA kits were used to detect the expressions of AGEs, MDA, TNF-*α*, IL-1*β,* and IL-6 in serum and tissue supernatants of the heart and kidney according to the manufacturer's instructions. RIPA lysate with phosphatase inhibitor was used to extract proteins from heart and kidney tissues. The concentrations of these proteins were detected by BCA kit. Finally, western blotting was used to detect the protein expressions of JNK (1 : 1000; Cell Signaling Technology 9252), p-JNK (1 : 1000; Cell Signaling Technology 4668), p38 (1 : 1000; Cell Signaling Technology 8690), p-p38 (1 : 1000; Cell Signaling Technology 4511), ERK1/2 (1 : 1000; Cell Signaling Technology 4695), p-ERK1/2 (1 : 2000; Cell Signaling Technology 4370), and *β*-actin (1 : 1000; Cell Signaling Technology 4970) in heart and kidney tissues.

### 2.9. Statistical Analysis

SPSS 24.0 software (Inc., Chicago, IL, USA) was used to statistically analyze the experimental data in this study. All statistical analyses were performed using one-way ANOVA followed by the least significant difference (LSD) test or Dunnett's T3 test. All data were expressed as the mean ± standard deviation. Results were considered statistically significant when *p* < 0.05. GraphPad prism 8.0.1 (San Diego, CA) was used for image production.

## 3. Results

### 3.1. Active Compounds of YJHD

**Figure 2:**
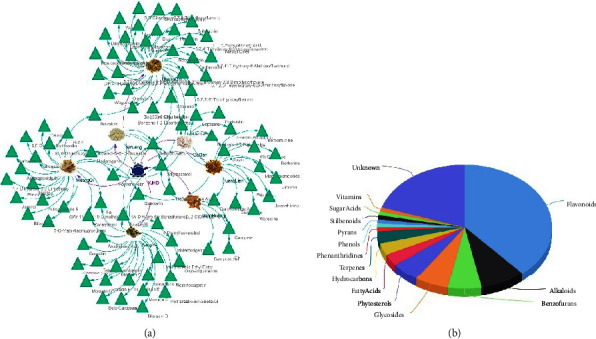
Analysis of YJHD compounds. (a) YJHD-Herbs-Compounds network. (b) Compound types of YJHD.

A total of 96 compounds were identified from YJHD based on TCMSP, PubMed, and UHPLC. OB indicates the percentage of oral drug reaching the systemic circulation. High OB is often a key indicator for oral therapeutics screening [33]. In this study, OB ≥ 30% was used as the screening standard for compounds in TCMSP. Among them, 17 compounds have OB > 60%, which have the basic characteristics and great potential to be developed into oral drugs. DL is the similarity of a compound to a known drug. Compounds with high DL have a greater possibility of becoming drugs [15]. In traditional Chinese medicine studies, DL is usually set at ≥0.18, which is also the compound screening criterion adopted in this study. HL refers to the time taken for the amount of compound in the body to fall by half, which determines the duration of the therapeutic effect that a compound can exert, HL ≤ 4 hours for rapid elimination, 4∼8 hours for intermediate elimination, and ≥8 hours for slow elimination [34]. HL > 4 hours was used as one of the compound screening criteria in this study. In addition, 47 of all 96 compounds had HL ≥ 8 hours. The YJHD-Herbs-Compound network is shown in Figure 2(a). Notably, 37 of the all 96 compounds are flavonoids, accounting for 38.5% of the total number of compounds (Figure 2(b)). Flavonoids have been shown to be beneficial in diabetes and diabetic complications [35, 36].

### 3.2. Putative Targets of YJHD

**Figure 3:**
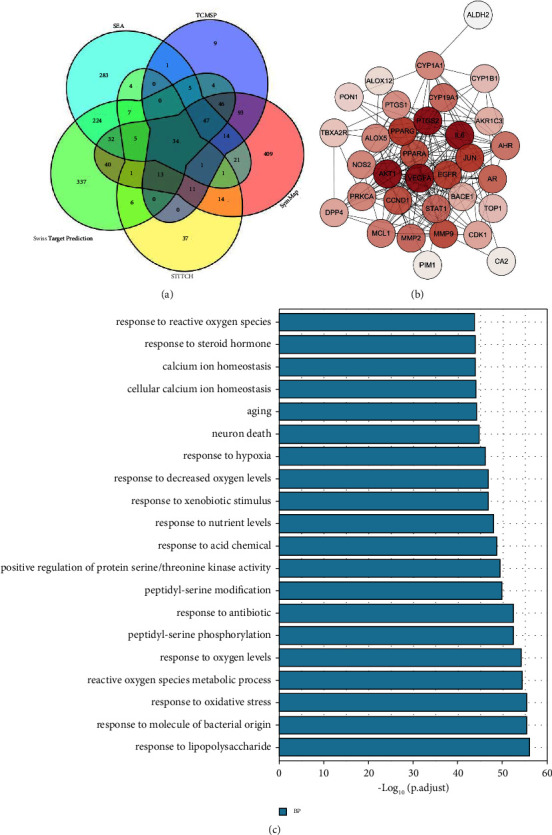
Collection and analysis of the YJHD targets. (a) Sources of the YJHD targets. (b) Targets commonly contained in 5 different databases. (c) Results of GO enrichment analysis of the YJHD targets (biological process).

Based on TCMSP, SymMap, STITCH, Swiss Target Prediction, and SEA databases, there are 1,699 targets of YJHD which were extracted (Figure 3(a)). Different databases contained varying numbers of YJHD targets, of which 34 targets are common to the five databases (Figure 3(b)). Among all the YJHD targets, a total of 1,649 targets were identified by the STRING database. Based on the information from STRING database, PPI networks of the YJHD targets were constructed. In PPI network, the median target Degree was 39. In addition, 420 targets had Degree ≥78. The top 10 targets in terms of Degree were AKT1, GAPDH, TP53, ALB, TNF, IL6, INS, CTNNB1, SRC, and MYC. The DAVID platform was used for the enrichment of the YJHD targets. A total of 601 of the YJHD targets were enriched under the disease item Type 2 Diabetes. In addition, BP items were extracted from the results of the GO enrichment analysis. The top 20 BP are shown in Figure 3(c).

### 3.3. Target Genes for Diabetic Microvascular Complications

**Figure 4:**
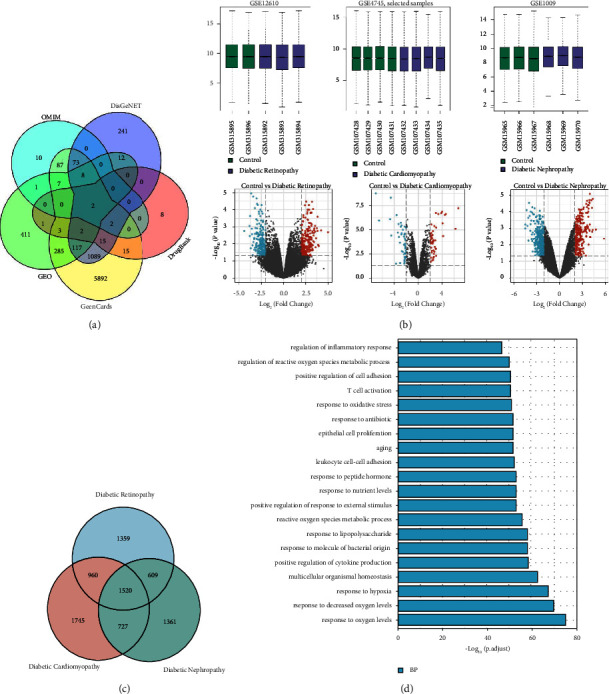
Collection and analysis of diabetic microvascular complications target genes. (a) Source of target genes. (b) High-throughput data of DR, DCM, and DN in GEO database. (c) Common target genes of DR, DCM, and DN. (d) GO enrichment analysis (biological process) of common target genes of DR, DCM, and DN.

Based on GeneCards, DrugBank, DisGeNET, OMIM, and GEO databases, a total of 8,281 target genes of diabetic microvascular complications were extracted (Figure 4(a)). It contained 4,448 target genes for DR, 4,952 target genes for DCM, and 4,217 target genes for DN. In the GEO database, high-throughput sequencing data for DR (GSE12610), DCM (GSE4745), and DN (GSE1009) were extracted (Figure 4(b)). These data consisted of 849 differentially expressed genes for diabetic microvascular complications. Based on the Venn diagram, 1,520 common target genes of DR, DCM, and DN were extracted (Figure 4(c)). These common target genes were entered into the DAVID platform for GO enrichment. BP items were extracted from the results of GO enrichment. The top 20 BP are presented in Figure 4(d).

### 3.4. BP-Based Analysis of Treatment Mechanisms

**Figure 5:**
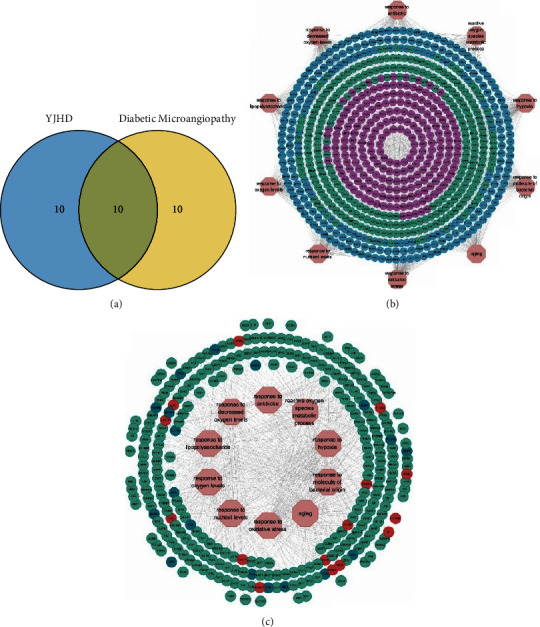
BP-based analysis of therapeutic mechanisms. (a) Intersection of BP items for YJHD targets and diabetic microvascular complications target genes. (b) Targets-BP network. Blue indicates the YJHD targets, purple indicates target genes of diabetic microvascular complications, and green indicates the intersection of the YJHD targets and diabetic microvascular complication target genes. (c) Intersection targets-BP network. Red indicates high expression genes and blue indicates low expression genes.

The first 20 BP of YJHD and the first 20 BP of diabetic microvascular complications were taken as intersections. Based on the Venn diagram, 10 overlapping BP items were extracted (Figure 5(a)). These items were related to oxidative stress, cellular energy metabolism, and immune response. There were 527 YJHD targets and 502 target genes for diabetic microvascular complications enriched on these 10 BP entries (Figure 5(b)). Of these, 277 were identified as intersecting targets for YJHD and diabetic microvascular complications (Figure 5(c)). These intersecting targets are the bridge for YJHD to exert influence on the biological processes and pathological phenotypes of diabetic microvascular complications. Among these intersecting targets, 33 targets were identified as differentially expressed genes for diabetic microvascular complications (Figure 5(c)).

### 3.5. Hub Genes of YJHD for Diabetic Microvascular Complications

**Figure 6:**
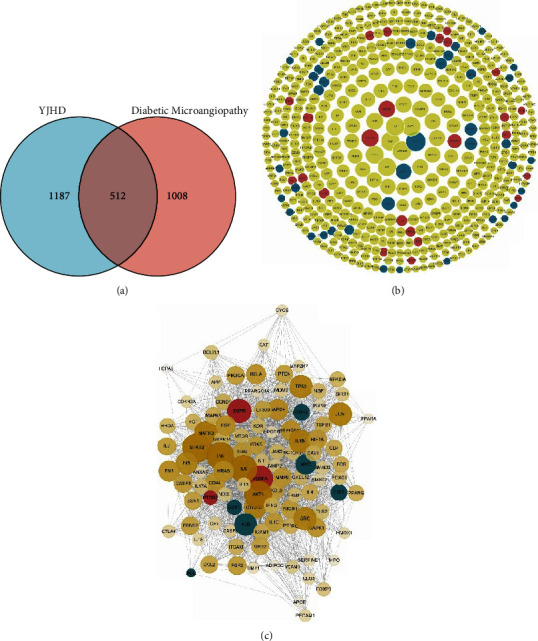
Hub gene screening. (a) Intersection of the YJHD targets and target genes for diabetic microvascular complications. (b) PPI network of intersecting targets. (c) Hub genes of YJHD for diabetic microvascular complications. Red indicates high expression genes and blue indicates low expression genes.

Based on the Venn diagram, 1,699 targets of YJHD and 1,520 common target genes of diabetic microvascular complications were taken to intersect. A total of 512 targets were extracted (Figure 6(a)). Among these, 509 targets were identified by STRING database. Based on the information provided by STRING database, the PPI network of intersecting targets was constructed (Figure 6(b)). In the PPI network, target Degree had a maximum of 336, a minimum of 1, and a median of 56. Among them, 105 targets had Degree ≥112, which were identified as the Hub genes of YJHD for the treatment of diabetic microvascular complications (Figure 6(c)). Nine Hub genes were identified as differentially expressed genes for diabetic microvascular complications by taking intersection between Hub genes and differentially expressed genes of diabetic microvascular complications.

### 3.6. Hub Gene-Based Enrichment Analysis

**Figure 7:**
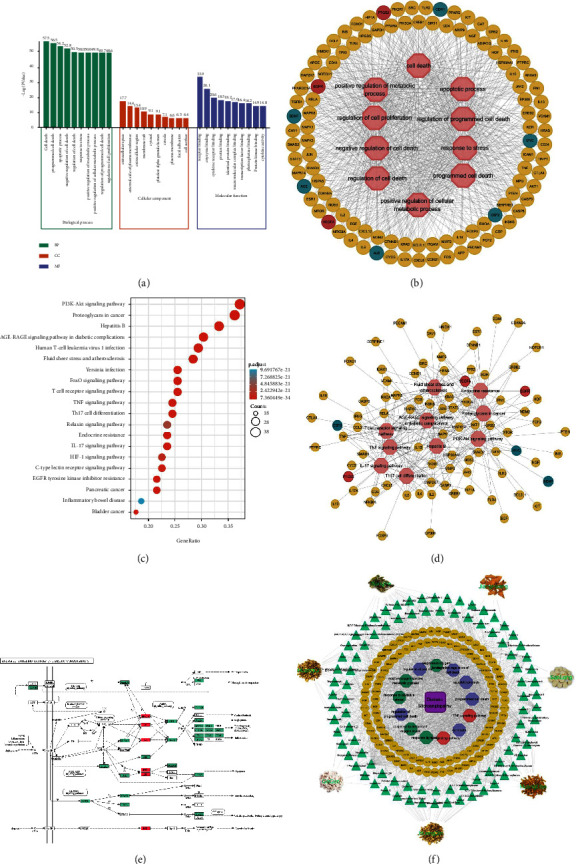
Enrichment analysis of Hub genes. (a) Results of GO enrichment analysis of Hub genes. (b) Hub genes-BP network. (c) Results of the KEGG enrichment analysis of Hub genes. (d) Hub genes-pathways network. (e) Mapping of Hub genes in the AGE-RAGE signaling pathway in diabetic complications item. (f) Herbs-Compounds-Hub genes-BP/pathways-Disease network.

Hub genes were entered into the DAVID platform for GO enrichment analysis. The results of GO enrichment included three parts, BP, molecular function (MF), and cellular component (CC). The top 10 items of BP, MF, and CC are shown in Figure 7(a). Hub genes are widely expressed in the extracellular space, cell membrane, and cytoplasm and are associated with the binding of various cytokines, receptors, and enzymes. Based on the abovementioned biological properties, Hub genes are involved in regulating various biological processes such as cell proliferation, apoptosis, and metabolism. Notably, all 105 Hub genes were enriched in the top 10 BP items (Figure 7(b)).

The STRING database was used for the KEGG pathway enrichment. A total of 102 Hub genes were identified by STRING database. The top 20 KEGG pathways are shown in Figure 7(c). Among them, the PI3K/Akt signaling pathway has the most enriched genes with 38. The AGE-RAGE signaling pathway in diabetic complications has the smallest p adjust value and also has 31 Hub genes. In addition, several signaling pathways are associated with cellular energy metabolism, immune, and inflammatory response. 83 Hub genes are enriched in the top 10 KEGG pathways, which together form the Hub genes-pathways network (Figure 7(d)).

In the KEGG platform, the specific role of Hub genes in the AGE-RAGE signaling pathway was demonstrated by pathway mapping (Figure 7(e)). Notably, JNK, p38, and ERK1/2 play central signaling roles in a variety of biological functions mediated by the AGE-RAGE signaling pathway. Based on the integration of the abovementioned information, the Herbs-Compounds-Hub genes-BP/pathways-Disease network was constructed (Figure 7(f)).

### 3.7. Molecular Docking

**Figure 8:**
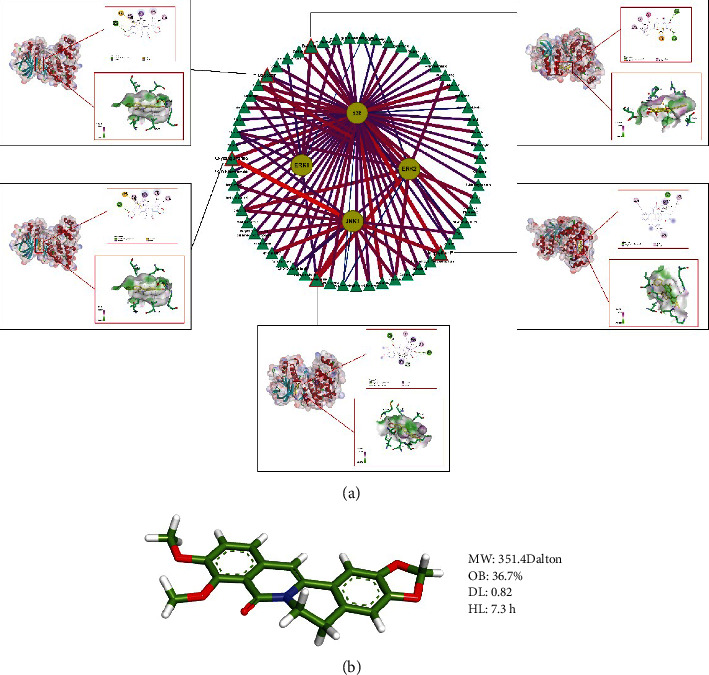
Molecular docking results. (a) Compounds-target proteins molecular docking network. The color and thickness of the edges in the diagram indicate the magnitude of the binding energy. The redder the color of the edge, the thicker the edge means the greater the binding energy. The five docking pattern diagrams show the docking sites of compounds with maximum binding energy to JNK, p38, ERK1, and ERK2. (b) 3D structure and ADME information of berlambine.

Autodock Vina software was used for molecular docking. 59 compounds were extracted from the Herbs-Compounds-Hub genes-BP/pathways-Disease network. These compounds constitute 93 correspondences with JNK1, p38, ERK1, and ERK2. Based on the correspondence between compounds and target proteins, the Compounds-Target proteins molecular docking network was constructed (Figure 8(a)). The binding between compounds and target proteins is considered stable when binding energy < −5 kcal/mol. Among all 93 docking relations, the binding energy was as low as −5.4 kcal/mol and as high as −10.5 kcal/mol. In addition, JNK1, p38, ERK1, and ERK2 had the highest binding energy with oxysanguinarine (−10.5 kcal/mol), inophyllum *E* (−9.7 kcal/mol), calycosin (−8.4 kcal/mol), baicalein (−8.4 kcal/mol), and berlambine (−9.6 kcal/mol), respectively. Among them, calycosin and baicalein had the same binding energy with ERK1, both at −8.4 kcal/mol (Figure 8(a)). Notably, berlambine targeted both JNK1 (−9.5 kcal/mol), p38 (−8.8 kcal/mol), ERK1 (−7.6 kcal/mol), and ERK2 (−9.6 kcal/mol), and all had binding energies < −7 kcal/mol. Therefore, berlambine may be a potential regulator of the MAPK protein family. The 3D structure of the berlambine molecule is shown in Figure 8(b). Discovery Studio software was used to visualize the molecular docking results.

### 3.8. YJHD Improves the Pathological Changes of Diabetic Microvascular Complications

**Figure 9:**
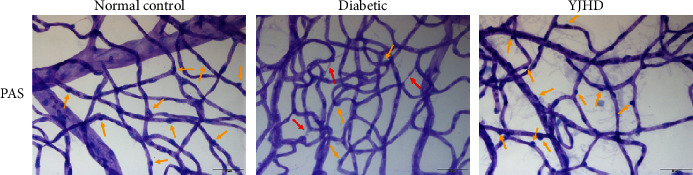
PAS staining of retinal capillaries. Yellow arrows indicate pericytes and red arrows indicate neocapillaries.

In previous studies, we confirmed the therapeutic effect of YJHD on DN and DCM [12, 13]. In the kidney of diabetic rats, YJHD ameliorated pathological changes such as renal enlargement, mesangial area expansion, extracellular matrix accumulation, basement membrane thickening, and podocyte injury caused by diabetes [12]. In the heart tissue of diabetic rats, YJHD ameliorated pathological changes such as cardiomyocyte hypertrophy, increased intracellular fat vacuolization, myocardial fiber disarray, and fibroplasia induced by diabetes [13]. To assess the effect of YJHD on DR, PAS staining was used to examine the vascular system of the retina in diabetic rats. Pericyte loss is one of the early features of DR and is associated with retinal vascular damage [37]. Compared with the normal control group, the retinal vascular system in the diabetic group rats showed an increase in acellular neocapillaries and a decrease in the number of pericytes. YJHD inhibited acellular capillary angiogenesis and alleviated the decrease in pericyte number compared to the diabetic group (Figure 9).

### 3.9. Effects of YJHD on Inflammation and Oxidative Stress

Based on network pharmacology results, inflammatory response, oxidative stress, and apoptosis were identified as key pathological phenotypes for YJHD in the treatment of diabetic microvascular complications. The inflammatory response, oxidative stress, and apoptosis interact with each other and together lead to the development and progression of diabetic microvascular complications [38, 39]. The inflammatory response and oxidative stress induced by diabetes are key factors leading to apoptosis. In the present study, the effects of YJHD on inflammatory response and oxidative stress in diabetic rats were evaluated.

**Figure 10:**
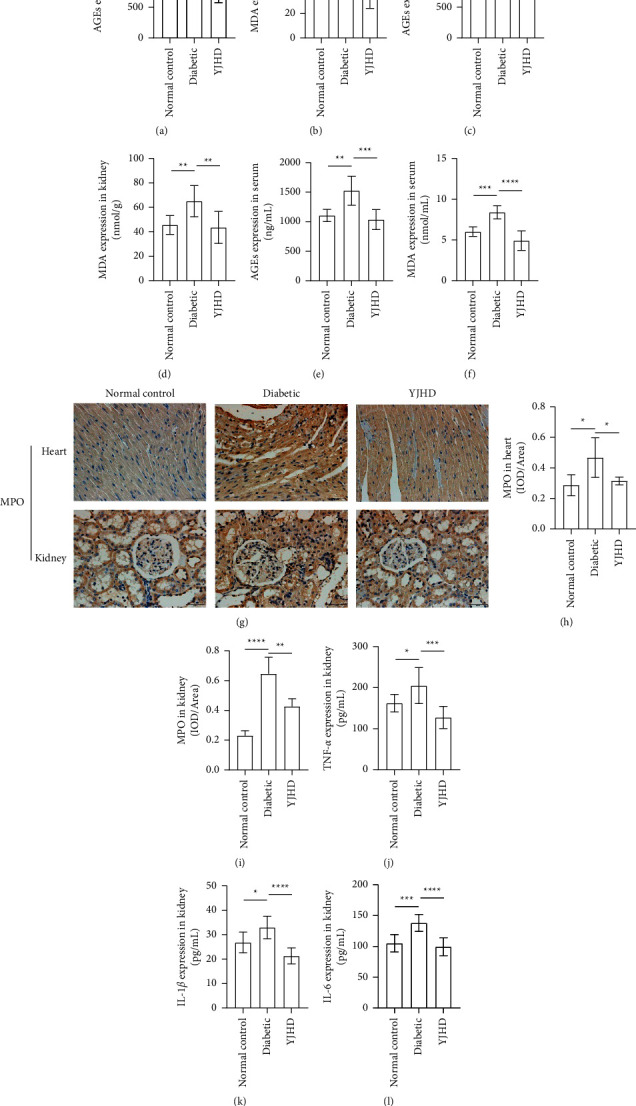
Effect of YJHD on inflammation and oxidative stress. (a)–(f) Expression of AGEs and MDA in the heart, kidney, and serum. (g)–(i) Expression of MPO in the heart and kidney. (j)–(l) Expression of TNF-*α*, IL-1*β,* and IL-6 in the kidney. ^*∗*^*P* < 0.05, ^*∗∗*^*P* < 0.01, ^*∗∗∗*^*P* < 0.001 and ^*∗∗∗∗*^*P* < 0.0001.

The levels of AGEs and MDA correlated with the degree of oxidative stress [40]. The expression levels of AGEs and MDA were significantly elevated in the heart, kidney, and serum of diabetic rats. AGEs and MDA were significantly decreased in the YJHD group compared with the diabetic group (Figures 10(a)–10(f)). This suggests that YJHD may improve oxidative stress in diabetic rats. MPO is rich in neutrophils. In inflammatory response, MPO is released by neutrophils and thus participates in the clearance of pathogens [41]. The increase of MPO content in heart and kidney of diabetic rats indicates the occurrence of inflammation. Compared with the diabetic group, YJHD decreased the content of MPO in the heart and kidney (Figures 10(g)–10(i)). TNF-*α*, IL-1*β,* and IL-6 are three proinflammatory cytokines [42]. Our previous studies demonstrated that YJHD was effective in reducing TNF-*α*, IL-1*β,* and IL-6 overexpression in the heart of diabetic rats. In this study, we also found increased expression of TNF-*α*, IL-1*β,* and IL-6 in the kidney tissue. The expression levels of TNF-*α*, IL-1*β,* and IL-6 in the rat kidneys of the YJHD group were significantly lower than those in the diabetic group (Figures 10(j)–10(l)). This suggests that YJHD inhibits inflammation in the heart and kidney of diabetic rats.

### 3.10. Effect of YJHD on MAPK Protein Family

**Figure 11:**
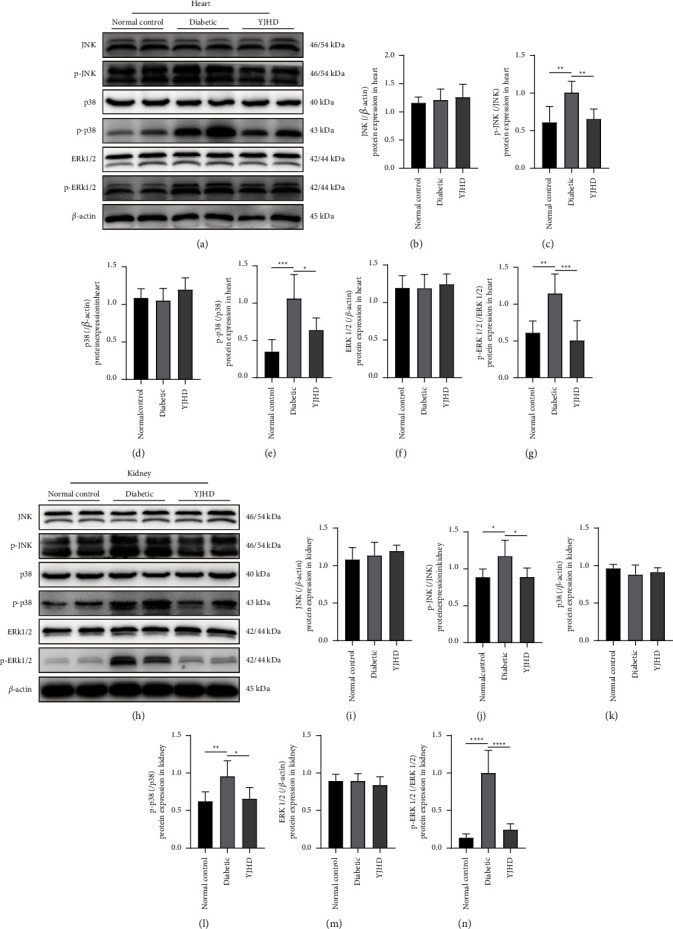
Effect of YJHD on MAPK protein family. (a)–(g) Effect of YJHD on the phosphorylation levels of JNK, p38, and ERK1/2 in rat heart tissues. (h)–(n) Effect of YJHD on the phosphorylation levels of JNK, p38, and ERK1/2 in rat kidney tissues. ^*∗*^*P* < 0.05, ^*∗∗*^*P* < 0.01, ^*∗∗∗*^*P* < 0.001, and ^*∗∗∗∗*^*P* < 0.0001.

JNK, p38, and ERK1/2 in the MAPK protein family play central signaling roles in the AGE-RAGE signaling pathway and are the focus of this study. Western blotting was used to detect protein expression levels of JNK, p-JNK, p38, p-p38, ERK1/2, and p-ERK1/2 in the heart and kidney tissues. The expression levels of JNK, p38, and ERK1/2 in the heart and kidney of rats did not differ significantly between the groups. Compared with the normal control group, the expression levels of p-JNK, p-p38, and p-ERK1/2 in the kidney and heart of diabetic rats were increased to a different extent. This suggests that JNK, p38, and ERK1/2 are activated by phosphorylation. Compared with the diabetic group, the expression levels of p-JNK, p-p38, and p-ERK1/2 in the heart and kidney of YJHD-treated rats were significantly decreased (Figure 11). This suggests that YJHD inhibits phosphorylation of JNK, p38, and ERK1/2.

## 4. Discussion

Diabetic microvascular complications have complicated pathogenesis. Multiple factors are thought to be associated with the progression of diabetic microvascular complications. DR, DCM, and DN are three common diabetic microvascular complications [39, 43]. Based on five different databases, a total of 1,520 genes were identified as common target genes for DR, DCM, and DN. These genes hide the common pathogenesis of diabetic microvascular complications. As a traditional Chinese medicine compound composed of seven herbs, YJHD has complex chemical composition and active targets. Based on five different databases, 96 compounds met the screening criteria for this study, targeting 1,699 putative targets. By influencing these targets, YJHD is able to exert its possible pharmacological effects. Analysis of therapeutic mechanisms based on BP and Hub genes was used to elucidate the possible mechanisms of YJHD in the treatment of diabetic microvascular complications. The results of enrichment analysis consisted of multiple BP items and the KEGG pathways. They point to three key pathological phenotypes: oxidative stress, inflammatory response, and apoptosis. By cross-talk between the multiple signaling pathways, oxidative stress, inflammation, and apoptosis can affect each other [44]. Oxidative stress and inflammation are important inducers of apoptosis. Therefore, this study focused on the role of oxidative stress and inflammation.

Oxidative stress is a specific state in which the relative balance of oxidation and antioxidation in the body is tilted towards the oxidative side and is thought to play an important role in the development of diabetes and its complications [45, 46]. In the diabetic state, prolonged hyperglycemia and hyperlipidemia can cause a substantial production of reactive oxygen species (ROS) in mitochondria or cytoplasm, thereby activating oxidative stress [47]. A large number of oxidative intermediates caused by persistent oxidative stress damage the bioactive molecules of cells, including proteins, lipids, and DNA [48, 49]. The impairment of these bioactive molecules is associated with pancreatic islets *β* cells damage, insulin signaling dysfunction, cell apoptosis, and tissue inflammation [50]. The abnormal state of these tissues and cells plays an important role in the development of diabetes and its complications. In this study, the Hub genes were enriched in several BP items associated with oxidative stress. These items include response to oxidative stress and reactive oxygen species metabolic process. In the diabetic state, oxidative stress promotes the production of AGEs. Large amounts of AGEs activate the AGE-RAGE signaling pathway by binding to RAGE receptors, further promoting high expression of RAGE, which in turn increases tissue damage due to oxidative stress [51]. In this study, AGEs and MDA were used to evaluate oxidative stress levels. We observed significantly elevated levels of AGEs and MDA in the heart, kidney, and serum of diabetic rats, suggesting the occurrence of oxidative stress. YJHD improves oxidative stress in these tissues, consistent with our network pharmacological prediction.

Inflammation plays an important role in the occurrence and progression of many chronic diseases. The shadow of inflammation can be seen in both diabetes and diabetic complications [52]. In the persistently high glucose state, glucose polymerizes with protein, resulting in large amounts of AGEs. Overexpressed AGEs specifically bind to RAGE receptors present on the surface of fibroblasts, smooth muscle cells, endothelial cells, monocytes/macrophages, and lymphocytes, activate NF-*κ*B through a series of intracellular signaling [53, 54]. Activated NF-*κ*B promotes transcription of multiple genes including endothelin-1, VCAM-1, ICAM-1, E-selectin, thrombomodulin, TF, vascular endothelial growth factor, IL-1, IL-6, TNF-*α,* and RAGE. High expression of these cytokines induces an inflammatory response and procoagulant state leading to vascular damage [54]. In this study, Hub genes were enriched in multiple KEGG pathways associated with inflammatory responses. In the AGE-RAGE signaling pathway, YJHD may not target the RAGE receptor, but exerts an influence on the signal transduction process after activation of RAGE. YJHD target NF-*κ*B, its upstream signaling proteins and downstream inflammatory cytokines. This may be the way it interferes with the inflammatory response. MPO is a protein rich in neutrophils. In inflammatory response, MPO is released by neutrophils and thus participates in the clearance of pathogen [41]. The level of MPO expression correlates with the degree of inflammatory response. TNF-*α*, IL-1*β,* and IL-6 are three inflammatory cytokines involved in inducing and promoting inflammation [42]. Our previous study demonstrated that YJHD was effective in reducing TNF-*α*, IL-1*β,* and IL-6 overexpression in the heart of diabetic rats [13]. In this study, we observed elevated expression levels of MPO in the heart and kidney of diabetic rats. In addition, TNF-*α*, IL-1*β,* and IL-6 were also elevated in kidney tissues of diabetic rats. After YJHD treatment, MPO in the heart and kidney, and TNF-*α*, IL-1*β,* and IL-6 in the kidney of rats all showed different degrees of reduction. This suggests that YJHD can effectively improve the inflammatory response in heart and kidney of diabetic rats. This result is consistent with the prediction of network pharmacology.

Apoptosis is one of the mechanisms of islet *β* cell destruction in both T1DM and T2DM and is thought to be associated with the occurrence of diabetes and its complications [55]. In addition, long-term hyperglycemia causes increased production of ROS, and a large number of ROS activates oxidative stress, further inducing islet *β* cell apoptosis, leading to aggravation of diabetes. Except to destroying islet *β* cells, apoptosis due to diabetes causes damage to the retina, heart, and kidney [56]. Inhibition of apoptosis has been confirmed to be associated with improvements in DR, DCM, and DN [57]. In this study, Hub genes were enriched in multiple BP items associated with apoptosis. These items include apoptotic process, programmed cell death, and regulation of programmed cell death. This suggests that apoptosis may be the key to YJHD in the treatment of diabetes and diabetic complications.

**Figure 12:**
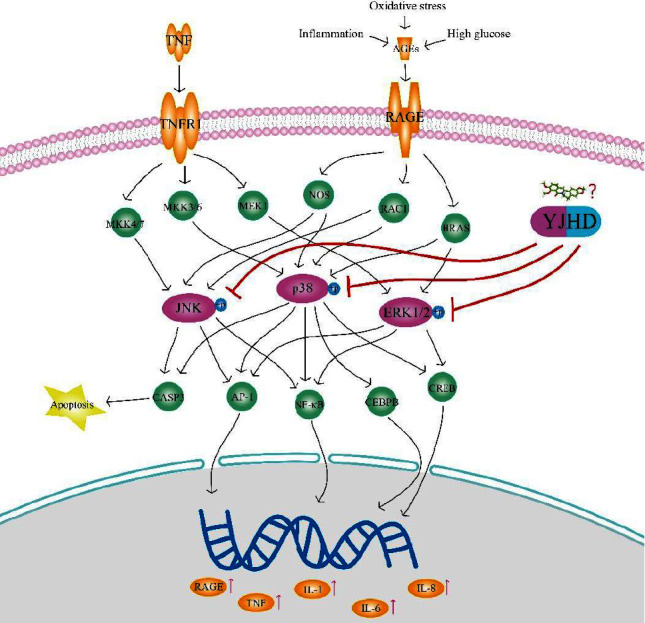
Possible mechanisms of YJHD for diabetic microvascular complications.

MAPK is a highly conserved protein class in eukaryotes. Human MAPK consists of four classical protein subfamilies, including ERK1/2 (MAPK3/1), p38 (MAPK 11-14), JNK (MAPK 8-10), and ERK5 (MAPK7). The MAPK protein family is associated with a variety of biological processes in cells [58]. Studies have shown that the MAPK protein family is activated by phosphorylation in the development of diabetes, which may be associated with tissue injury induced by diabetes [59]. In this study, the compounds in YJHD target JNK, p38, and ERK1/2 of the MAPK protein family. Notably, JNK, p38, and ERK1/2 play a central signaling role in the AGE-RAGE signaling pathway mediated diabetic complications. As shown in Figure 12, hyperglycemia, oxidative stress, and inflammation caused by diabetes increase the production of AGEs. A large number of AGEs bind to RAGE receptors and further induce and exacerbate oxidative stress, inflammatory response, and cell apoptosis by activating or upregulating the expression of RAGE, TNF-*α*, IL-1, IL-6, IL-8, and Caspase 3 [51]. This process requires the participation of JNK, p38, and ERK1/2. This means that JNK, p38, and ERK1/2 may be key therapeutic targets for blocking AGEs-induced tissue damage. This is the reason why the MAPK protein family is attracting increasing attention in the study of diabetes and diabetic complications. In this study, we observed increased phosphorylation of JNK, p38, and ERK1/2 in the heart and kidney of diabetic rats. In addition, YJHD inhibited phosphorylation activation of JNK, p38, and ERK1/2 to varying degrees. This suggests that the ameliorative effects of YJHD on oxidative stress and inflammatory response, as well as the potential regulatory effects on apoptosis, may be achieved by inhibiting phosphorylation of JNK, p38, and ERK1/2.

The results of molecular docking showed that the compounds in YJHD had high affinity to JNK, p38, ERK1, and ERK2, and stable binding could occur. This is the basis for YJHD to play its pharmacological role. Notably, where Notably, berlambine targets JNK, p38, ERK1, and ERK2, and all have binding energies < −7 kcal/mol. Berlambine is an alkaloid with molecular weight of 351.4 Dalton and is mainly rich in Coptis chinensis. According to Lipinski's rule of five, compounds with a molecular weight of 180–500 Dalton have the potential to become oral drugs in humans [60]. In addition, OB and DL of berlambine were 36.7% and 0.82, which means that berlambine has great potential to become an oral drug. HL for berlambine is 7.3 hours, which means friendly dosing frequency for patients. All of these properties suggest that berlambine has the potential to become an oral drug. Therefore, berlambine is considered a promising regulator of the MAPK protein family. More specific effects of berlambine on JNK, p38, ERK1, and ERK2 warrant were further studied.

Overall, this study predicted and evaluated the common mechanisms of YJHD in the treatment of DR, DCM, and DN. However, there are still some deficiencies in this study. 96 compounds that meet the parameters set in this study are considered to be the main therapeutic component of YJHD, but they are not strictly representative of YJHD. In this study, network pharmacology was used to predict the mechanism of YJHD in the treatment of diabetic microvascular complications. Databases are the basis for all network pharmacology analyses. Due to the imperfection of the database, there is an inevitable deviation between the results of network pharmacology and the reality. This study minimizes the bias caused by database defects by integrating multiple databases. Molecular docking was used to validate the binding of compounds and proteins. In the process of molecular docking, compounds and proteins are placed in an ideal environment in which water molecules and charges are removed. However, unlike the ideal environment, the real situation is that compounds and proteins are in a complex environment of force and electric fields. These differences will inevitably lead to a discrepancy between the molecular docking results and the real situation.

## 5. Conclusions

YJHD may play a therapeutic role in diabetic microvascular complications including DR, DCM, and DN by regulating oxidative stress, inflammatory response, and apoptosis. The regulation of phosphorylation of JNK, p38, and ERK1/2 may be crucial for the therapeutic effects of YJHD. Berlambine may play a key role in the treatment and is a promising regulator of the MAPK protein family. Our results provide the basis for further research in the future.

## Data Availability

The datasets used to support this study are available from the corresponding authors upon request.

## Supplementary Materials

Supplementary Materials Supplementary 1: TIC diagram of UHPLC-QExactive-MS detection of YJHD granule aqueous solution.
