# Supplementary material for: Potential Mechanisms of Yiqi Jiedu Huayu Decoction in the Treatment of Diabetic Microvascular Complications Based on Network Analysis, Molecular Docking, and Experimental Validation
Source: Evid Based Complement Alternat Med. 2023 Feb 10;2023:5034687. doi: 10.1155/2023/5034687 (PMC11401727; doi:10.1155/2023/5034687)
Supplement: Supplementary Materials — Supplementary 1: TIC diagram of UHPLC-QExactive-MS detection of YJHD granule aqueous solution. [file 5034687.f1.pdf]

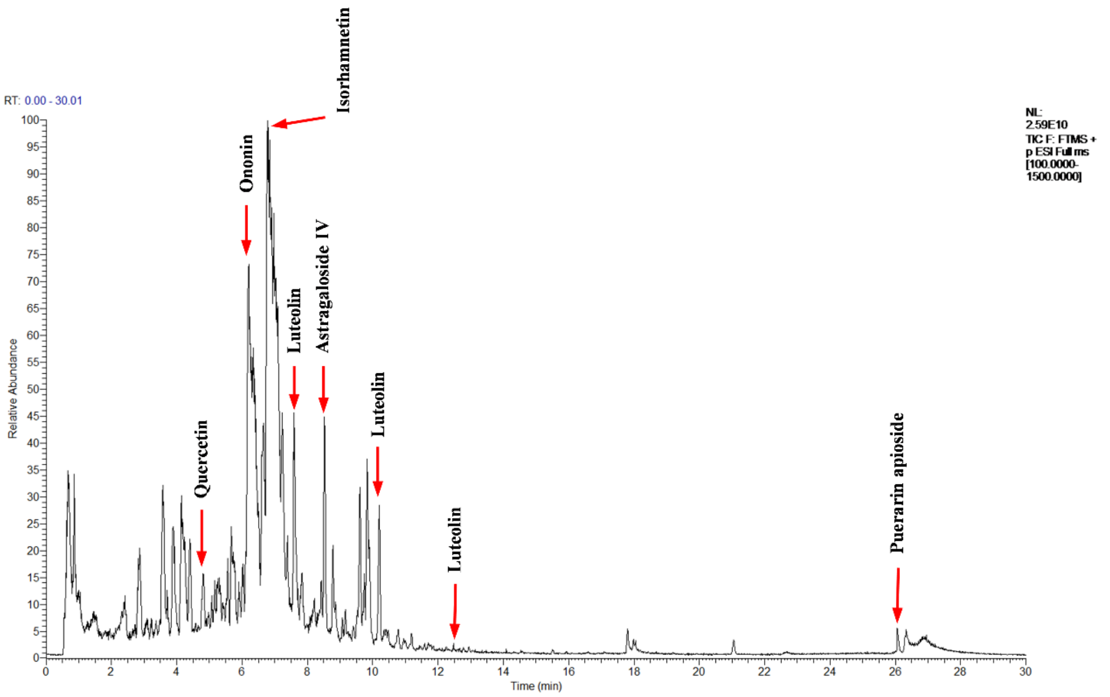

TIC diagram of UHPLC-Q Exactive-MS detection of YJHD granule aqueous solution in positive ion mode.

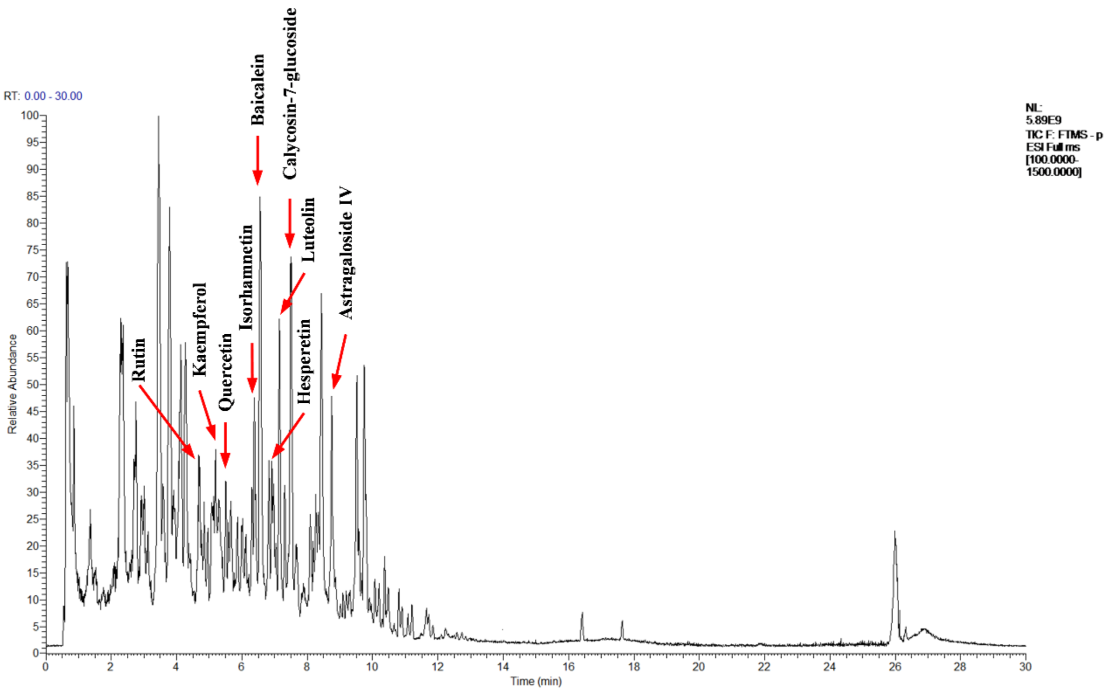

TIC diagram of UHPLC-Q Exactive-MS detection of YJHD granule aqueous solution in negative ion mode.
